# Comprehensive transcriptomic analysis reveals immune response modulation in *Brontispa longissima* Gestro larvae following parasitism by *Asecodes hispinarum* Bouček

**DOI:** 10.3389/fimmu.2026.1823349

**Published:** 2026-05-12

**Authors:** Zhiming Chen, Haiying Zhang, Jing Lin, Tingting Fan, Bo Cai, Yuanxiao Lin, Honghuai Zhang, Baozhen Tang, Youming Hou

**Affiliations:** 1 State Key Laboratory of Agricultural and Forestry Biosecurity, Fujian Agriculture and Forestry University, Fuzhou, China; 2 Integrated Technical Service Center of Rongcheng Customs, Fuzhou, China; 3 Animal and Plant Quarantine Center, Haikou Customs District, Haikou, China

**Keywords:** *Asecodes hispinarum*, *Brontispa longissima*, immune response, parasitism, transcriptome

## Abstract

**Introduction:**

The coconut leaf beetle, *Brontispa longissima*, is a major pest of palm plants in China and is significantly affected by the parasitoid wasp *Aseodes hispanarum*, which targets the beetle’s larval stage.

**Methods:**

Using single-molecule real-time (SMRT) sequencing, we constructed a high-quality transcriptome reference to assess gene expression associated with *A. hispanarum* parasitism in *B. longissima*. Differentially expressed genes (DEGs) were identified at 24, 48, 72, and 96 h post-parasitism compared with non-parasitized controls.

**Results:**

Based on 12,196 reference transcripts, a total of 2,120, 467, 500, and 5,749 DEGs were identified at 24, 48, 72, and 96 h post-parasitism, respectively. Over 75% of DEGs were upregulated across all time points. Gene Ontology (GO) enrichment analysis revealed significant enrichment of immune-related processes alongside hemocyte differentiation. Validation by quantitative PCR (qPCR) confirmed the consistency and reliability of the RNA-seq results.

**Discussion:**

The study identified immune-related genes differentially expressed in the fourth instar larvae of B. longissima. Overall, the results indicate that parasitism by *A. hispanarum* is associated with changes in the immune response of *B. longissima* larvae, offering preliminary insights into host-parasite interactions for potential biological control strategies.

## 1 Introduction

The coconut leaf beetle, *Brontispa longissima* Gestro (Coleoptera: Chrysomelidae), originally native to Indonesia and Papua New Guinea, has become a major invasive pest in China, particularly in the southern regions (1, 2). Both the larvae and adults of this beetle feed on the heart leaves of palm plants, causing significant damage such as leaf curling, wrinkling, and the wilting of tender shoots. In severe cases, this feeding can lead to the death of the entire plant (3). As a result, *B. longissima* has caused substantial economic losses to palm plantations in China (4, 5). Currently, both chemical and biological control methods are employed to manage this pest. The beetle typically feeds on the unexpanded heart leaves of its host, where the waxy coating on the leaves makes it difficult for pesticides to penetrate and effectively control the pest. Additionally, the height of some palm trees complicates pesticide application, further reducing the effectiveness of chemical control. Once introduced, the beetle is also difficult to eradicate (6, 7).

Biological control, particularly the use of parasitoids like *Asecodes hispinarum* Bouček (Hymenoptera: Eulophidae), has shown promise in managing *B. longissima*. *A. hispinarum*, a parasitic wasp native to Western Samoa and Papua New Guinea, targets the larvae of *B. longissima*, especially preferring the fourth instar larvae. It can also parasitize larvae and pupae of various instars when hosts are scarce (8–14). In March 2004, China introduced *A. hispinarum* from Vietnam for the biological control of *B. longissima*, and the results have been positive, as reported in several studies (7, 8, 11, 15).

Parasitic wasps like *A. hispinarum* regulate various physiological processes in their host insects, including immunity, metabolism, and development, ultimately leading to the host’s death. These wasps are vital natural control agents and play an effective role in pest management. While there is considerable knowledge on the effects of parasitism on *B. longissima* the specific impact on its immune system remains underexplored (2, 14, 16, 17). Third-generation transcriptome sequencing facilitates the acquisition of high-quality reference transcriptomes more easily than *de novo* reference-free transcriptome sequencing, making it highly valuable for studying gene expression changes (18–21). This study aims to use Illumina sequencing technology to obtain the transcriptome of *B. longissima* and analyze the differential expression of immune-related genes in both parasitized and non-parasitized larvae. The findings will offer valuable insights into how *A. hispinarum* parasitism affects the immune system of *B. longissima* and could contribute to the development of new pest control strategies targeting the pest’s immune response.

## 2 Materials and methods

### 2.1 Experimental insects

The *B. longissima* beetles and *A. hispinarum* wasps used in this experiment were sourced from the Environment and Plant Protection Institute at the Chinese Academy of Tropical Agricultural Sciences. *A. hispinarum* was reared on *B. longissima* larvae as hosts, and the adult wasps were provided with a 10% sucrose solution. The *B. longissima* larvae were fed leaves from *Washingtonia filifera* Linden.Wendland. Both species were maintained under controlled conditions at 25 ± 1 °C, with 70 ± 5% relative humidity and a 12-hour light/dark cycle.

The fourth instar larvae of *B. longissima* were placed in a petri dish with *A. hispinarum* to observe parasitism under a microscope. The larvae were then transferred to culture tubes with the appropriate amount of *Washingtonia filifera* palm leaves, and they were cultured under the same conditions. Samples were collected at 24, 48, 72, and 96 hours post-parasitism (PP), as well as for the control group (non-parasitized larvae, NP), to analyze gene expression at different time points. Samples were collected for each treatment and sent to Gene Denovo Biotechnology Co. (Guangzhou, China) for third-generation full-length transcriptome sequencing. Three independent biological replicates were included for each time point. Additionally, second-generation transcriptome sequencing was performed for comparison.

### 2.2 RNA extraction and transcriptome sequencing

The third-generation transcriptome library was constructed using the SMRTbell^®^ Prep Kit 3.0 and sequenced on the PacBio Sequel II platform. The SMRT Link V8.0.0 software was used to analyze the raw data. High-quality circular consensus sequences (CCS) were extracted, and poly(A) tails, primers, and barcodes were removed to obtain full-length non-chimeric (FLNC) reads. These reads were clustered using Minimap2, and the Quiver algorithm was applied to correct and retain high-quality transcripts. Redundant sequences were removed using cd-hit to obtain the final transcript sequence. Functional annotation of those high-quality transcripts were performed based on the NCBI non-redundant protein database, KEGG, InterPro, and Gene Ontology (GO) using DIAMOND BLASTX (E-value < 1e–10).

For second-generation sequencing, the Hieff NGS^®^ Ultima Dual-mode mRNA Library Prep Kit (12309ES, Yeasen) was used to construct the Illumina cDNA library, with a total of 24 libraries created. Sequencing was performed on the Illumina Novaseq 6000 platform. Raw reads were quality-controlled using Fastp, and low-quality data were filtered. Clean reads were then used for transcript quantification, and gene expression levels were estimated by RSEM based on the high-quality mapped reads.

### 2.3 Differential expression gene analysis

Differentially expressed genes (DEGs) between the parasitized and non-parasitized *B. longissima* larvae were identified based on the raw read counts generated by RSEM. And significantly DEGs were defined using thresholds of FDR ≤ 0.05 and |log_2_FC| ≥ 1. Gene Ontology (GO) enrichment analysis was performed using clusterProfiler, with adjusted p-values ≤ 0.05 considered significantly enriched. In addition, KEGG pathway analysis was performed to identify the major biochemical and signal transduction pathways associated with the DEGs.

### 2.4 qRT-PCR validation

Based on the differentially expressed genes from the transcriptome data, immune-related genes were screened and analyzed. These genes were further explored to understand how *A. hispinarum* parasitism influences the immune response of *B. longissima* larvae.

To validate the RNA-seq results, 12 genes were selected for qRT-PCR analysis. RNA samples were collected, and cDNA was synthesized using the Takara PrimeScript™ FAST RT Reagent Kit. qPCR was conducted using specific primers (**Supplementary Table 1**), and gene expression levels were calculated using the 2-ΔΔCt method.

## 3 Results

### 3.1 Analysis of SMAT sequencing results for B. longissima

#### 3.1.1 Length analysis of high quality transcripts in B. longissima

**Figure 1:**
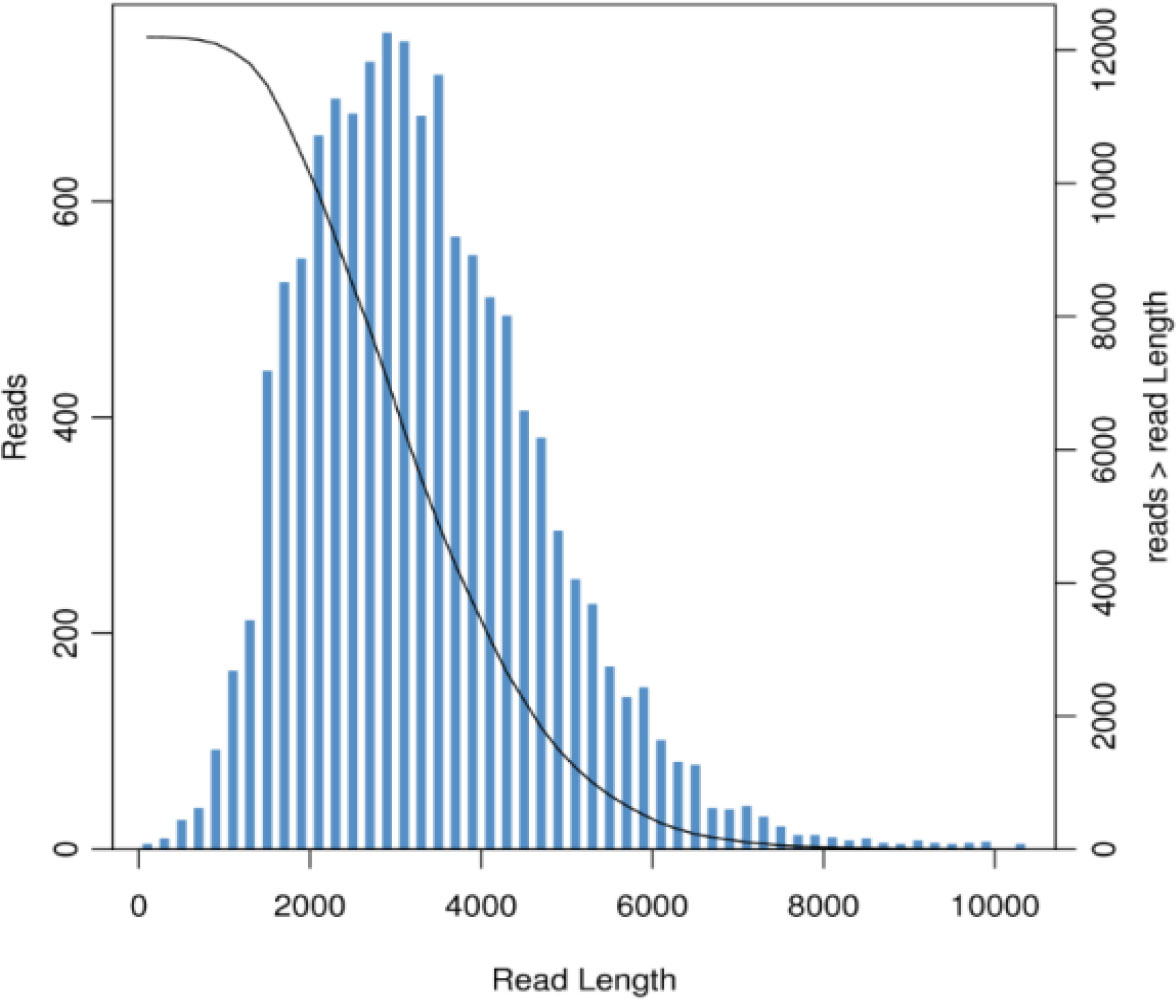
Length distribution of transcript sequences in the full-length transcriptome of *Brontispa longissima* larvae.The x-axis represents the length of the transcripts. The left y-axis corresponds to the histogram, showing the number of transcripts within each length interval along the x-axis. The right y-axis represents the cumulative distribution, indicating the number of transcripts with lengths greater than or equal to a given value on the x-axis.

After conducting single-molecule real-time (SMRT) sequencing, we obtained a total of 8,033,690 subreads, with an average length of 3,068 base pairs (bps), resulting in 24 gigabases (GB) of raw sequence data. Through the processes of clustering, error correction, and redundancy removal, we successfully generated the full-length transcriptome for B. longissima, yielding a total of 12,196 full-length transcript sequences. The longest transcript was 10,299 bps in length, while the average length of all transcripts was 3,310 bps (**Table 1**; **Figure 1**).

**Table 1:** Summary of single molecule real-time sequencing of *B. longissima*.

Sequencing Parameters	Number
Total base(bp)	24654271483
subreads number	8033690
average length	3068
N50	3404
Number of reads	229228
Number of CCS bases	783398881
CCS Read Length (mean)	3417
Number of Passes (mean)	29
Number of polished high-quality transcripts	13470
Number of polished low-quality transcripts	145
Total Number	12196
Total length (bp)	40378425
Maximum Length(bp)	10299
Minimum Length(bp)	104
Average Length(bp)	3310.79
N50 Length(bp)*	3716
GC content	39.48%

N50, Median length of all contigs or unigenes.

#### 3.1.2 transcripts functional annotation and classification for *B. longissima*

**Figure 2:**
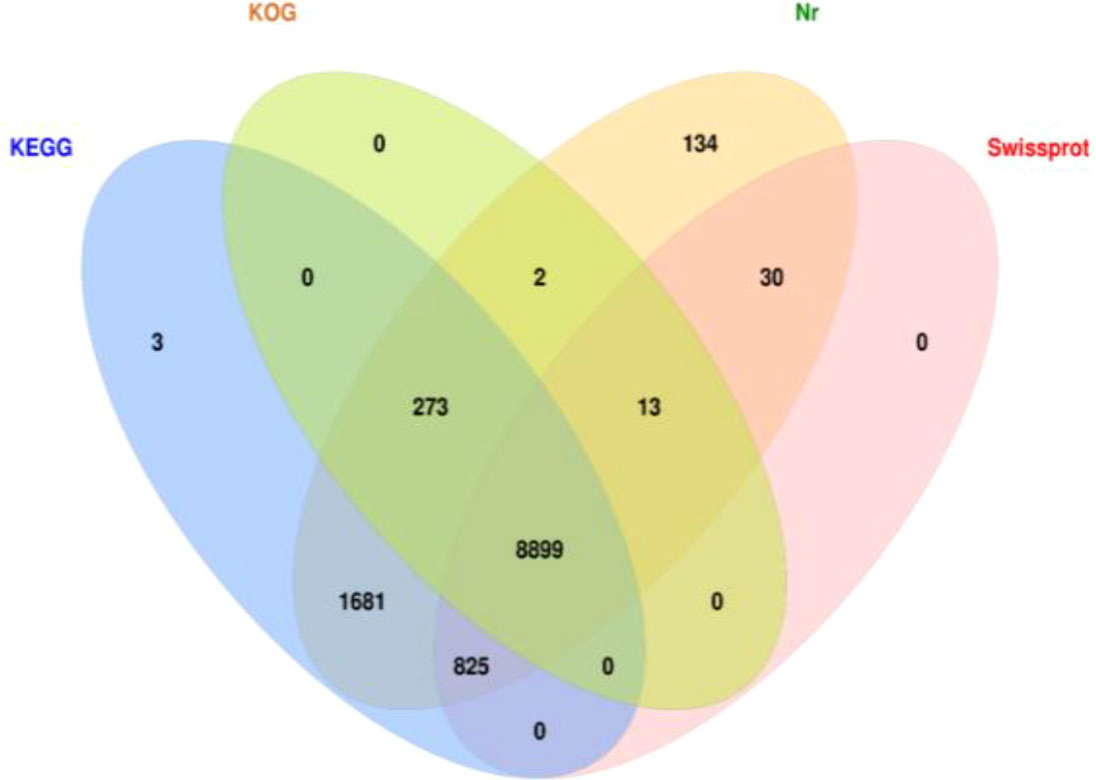
Functional annotation for the full-length transcriptome of *B. longissima* larvae. The diagram shows the overlap of transcripts annotated against four public databases: KEGG (blue), KOG (green), Nr (orange), and Swiss-Prot (pink). The numbers in each region represent the number of transcripts annotated in the corresponding database(s), with shared regions indicating transcripts annotated in multiple databases.

To assign functional annotations to the transcript sequences, we performed BLASTx comparisons with several protein databases, including Nr, SwissProt, KEGG, and COG/KOG (with an e-value threshold of < e-5). The top-ranked protein from each BLAST result was used to define the coding sequence (CDS) for each transcript. In total, 12,196 CDS sequences were identified, of which 11,860 transcripts were successfully annotated in at least one of the protein databases. However, 336 transcript sequences remained unannotated. Specifically, 11,857 transcripts were annotated in the NR database (**Figure 2**).

**Figure 3:**
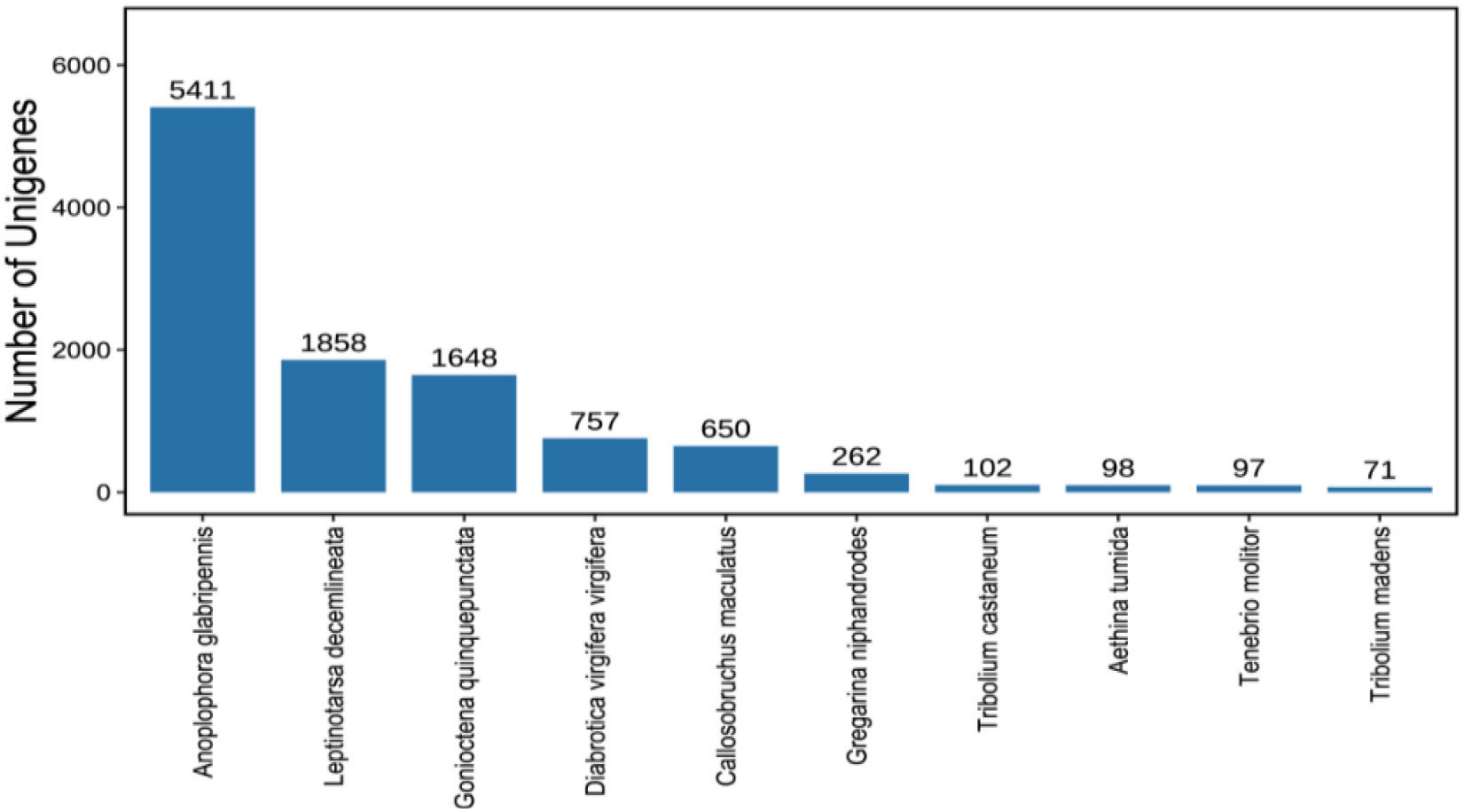
Nr-annotated species distribution statistics of the full-length transcriptome of (B) longissima larvae (Top 10). The X-axis represents the top 10 annotated species, while the Y-axis indicates the number of unigenes. Anoplophora glabripennis(5411 unigenes) showed the highest homology, followed by Leptinotarsa decemlineata(1858 unigenes) and Gonioctena quinquepunctata(1648 unigenes).

The functional annotation revealed that the species with the closest homology to *B. longissima* transcript sequences was Anoplophora glabripennis Motschulsky, another Coleopteran insect. In total, 5,411 transcripts of *A. glabripennis* were identified as closely related to *B. longissima* transcripts (**Figure 3**).

**Figure 4:**
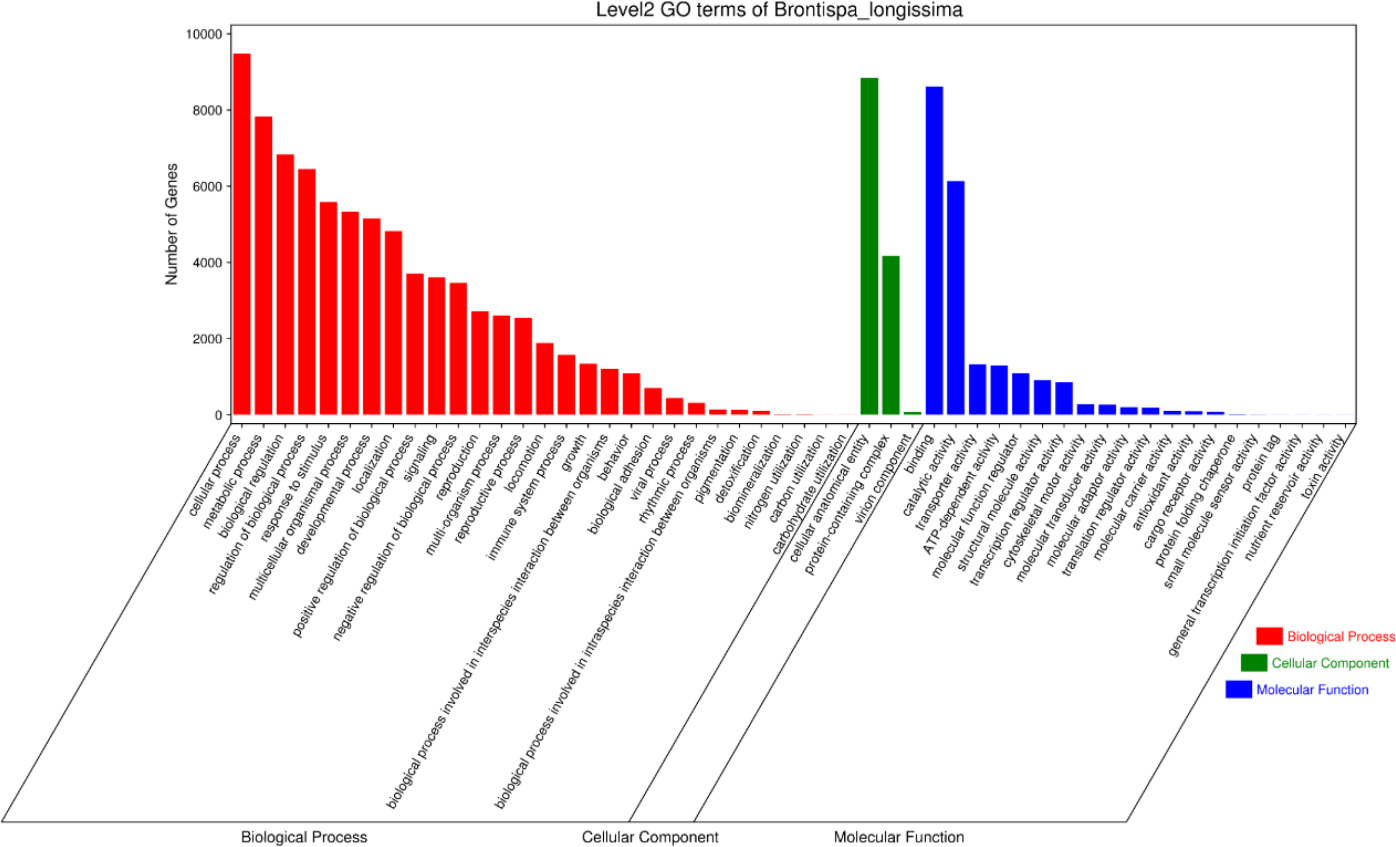
GO functional classification of the full-length transcriptome of (B) longissima larvae. The distribution of annotated unigenes is categorized into three main groups: Biological Process (red), Cellular Component (green), and Molecular Function (blue). The X-axis lists the specific Level 2 GO terms, and the Y-axis represents the number of genes associated with each term.

Gene Ontology (GO) analysis was performed to predict the potential functions of the inferred proteins. In total, 11,857 transcripts were successfully annotated with GO terms and classified into three main categories: Biological Process, Cellular Component, and Molecular Function. Within the Biological Process category, the largest proportion of genes was associated with cellular processes (9,475 genes) and metabolic processes (7,826 genes). In the Cellular Component category, most genes were annotated to cellular anatomical entities (8,846 genes), followed by protein-containing complexes (4,171 genes). For the Molecular Function category, the majority of genes were related to binding activity (8,611 genes), while a substantial number were involved in catalytic activity (6,130 genes) (**Figure 4**).

**Figure 5:**
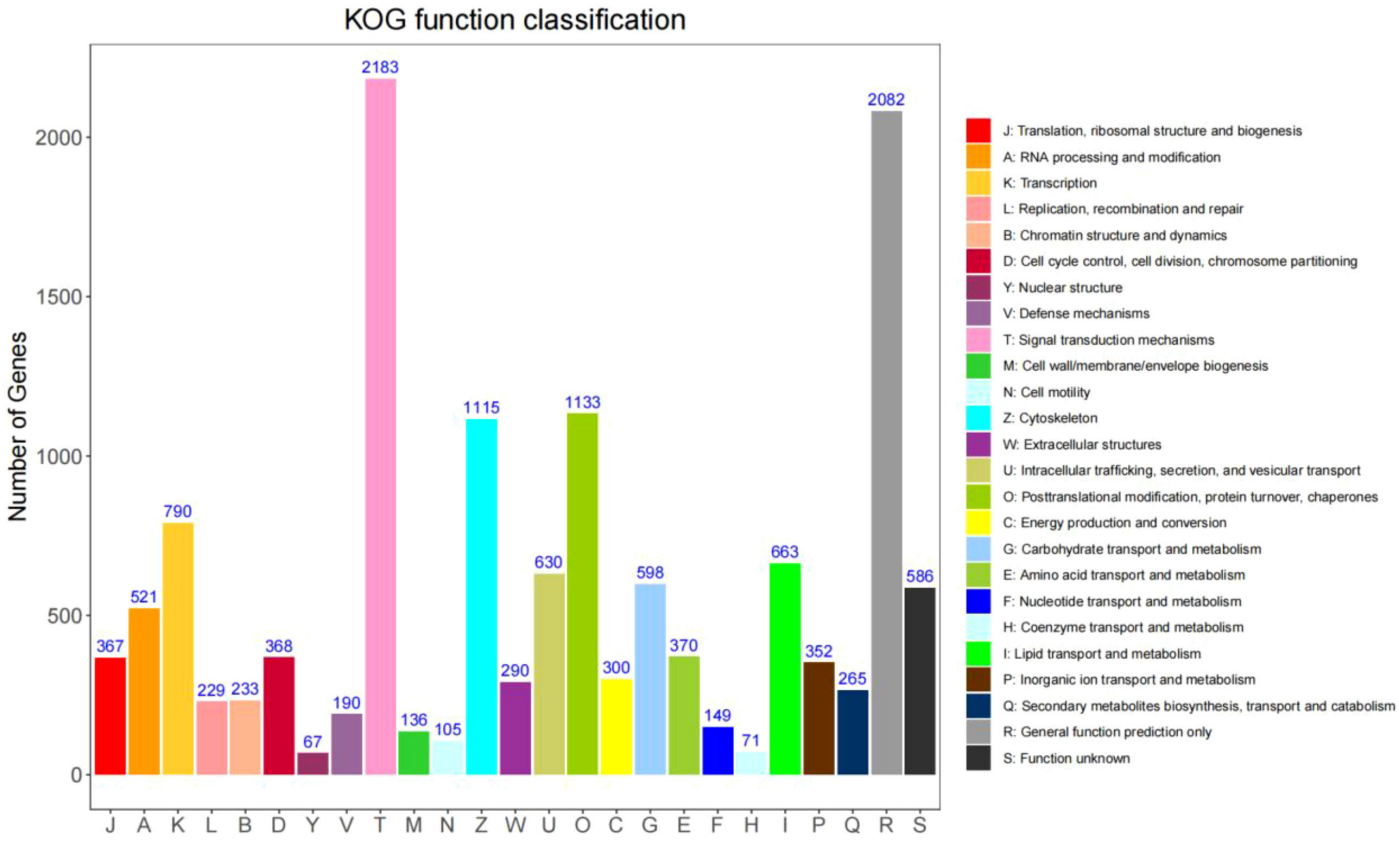
KOG functional classification of the full-length transcriptome of (B) longissima larvae. The X-axis represents the 24 KOG categories (abbreviated from A to S), while the Y-axis indicates the number of annotated unigenes. Different colors correspond to various functional categories, with the specific class represented by each color detailed in the legend on the right. Notably, the categories “General function prediction only” (R, grey bar) and “Signal transduction mechanisms” (T, pink bar) contain the highest number of unigenes, with 2082 and 2183 respectively.

To further characterize the functional roles of the predicted proteins, KOG analysis was also conducted. The annotated transcripts were grouped into 25 KOG functional categories. Among these, signal transduction mechanisms represented the most abundant category, comprising 2,183 transcripts. transcripts with unknown functions formed the second largest group, whereas nuclear structure was the least represented category, with only 67 transcripts (**Figure 5**).

### 3.2 Next-generation sequencing in *B. longissima*

#### 3.2.1 Transcriptome profiling of differentially expressed genes

**Figure 6:**
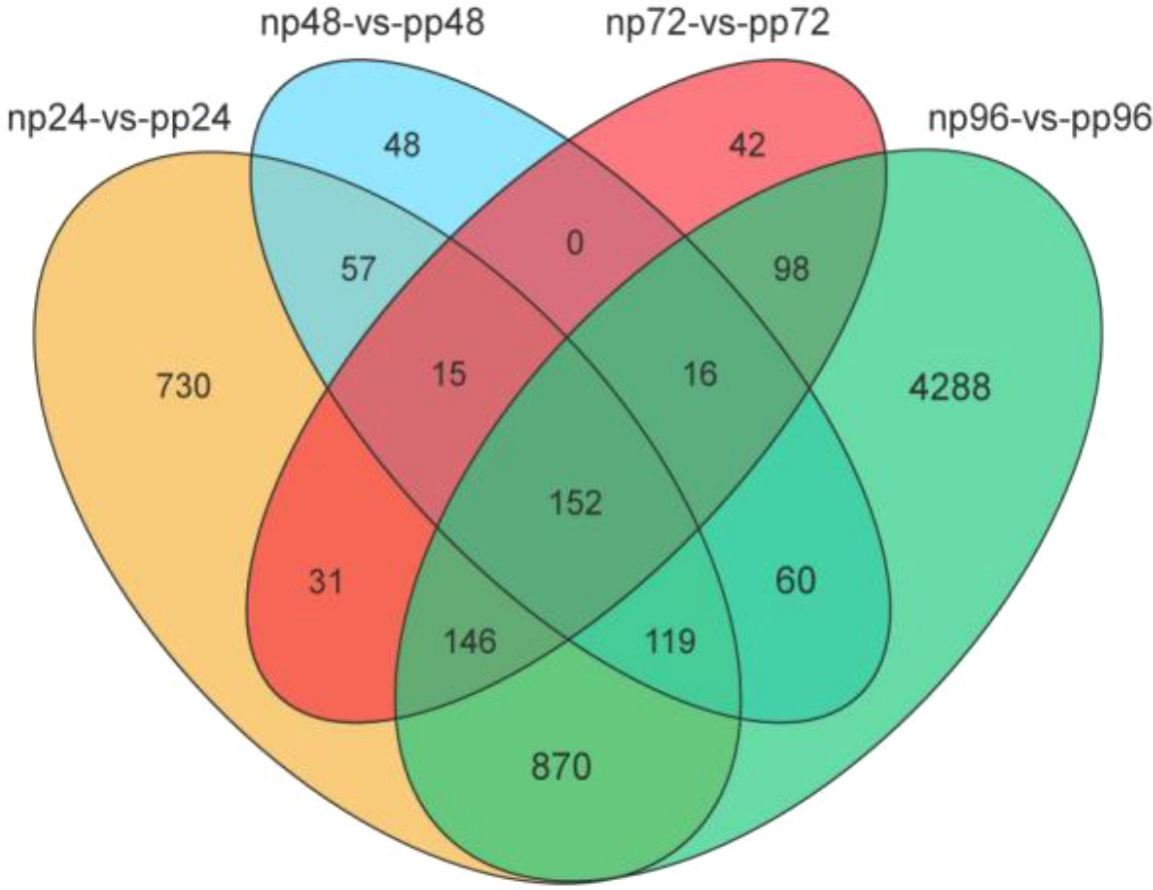
Venn diagram of DEGs identified at 24 h, 48 h, 72 h, and 96 h post parasitization of (B) longissima*larvae* by (A) hispinarum.The diagram illustrates the overlap of differentially expressed genes (DEGs) among the four time points. Each circle represents the unique and shared DEGs for a specific comparison group: np24-vs-pp24 (yellow), np48-vs-pp48 (light blue), np72-vs-pp72 (red), and np96-vs-pp96 (green). The numbers within each section indicate the count of DEGs specific to that group or shared between groups.

Based on the second-generation (NGS) sequencing data, transcript abundance was analyzed to identify genes that were differentially expressed between non-parasitized (NP) and parasitized (PP) larvae at different time points. Across the comparisons np24 vs. pp24, np48 vs. pp48, np72 vs. pp72, and np96 vs. pp96, a total of 6,672 differentially expressed genes (DEGs) were identified, of which 152 genes were shared among all four comparison groups. The numbers of DEGs uniquely expressed at 24 h, 48 h, 72 h, and 96 h post-parasitism were 730, 48, 42, and 4,288, respectively (**Figure 6**).

**Figure 7:**
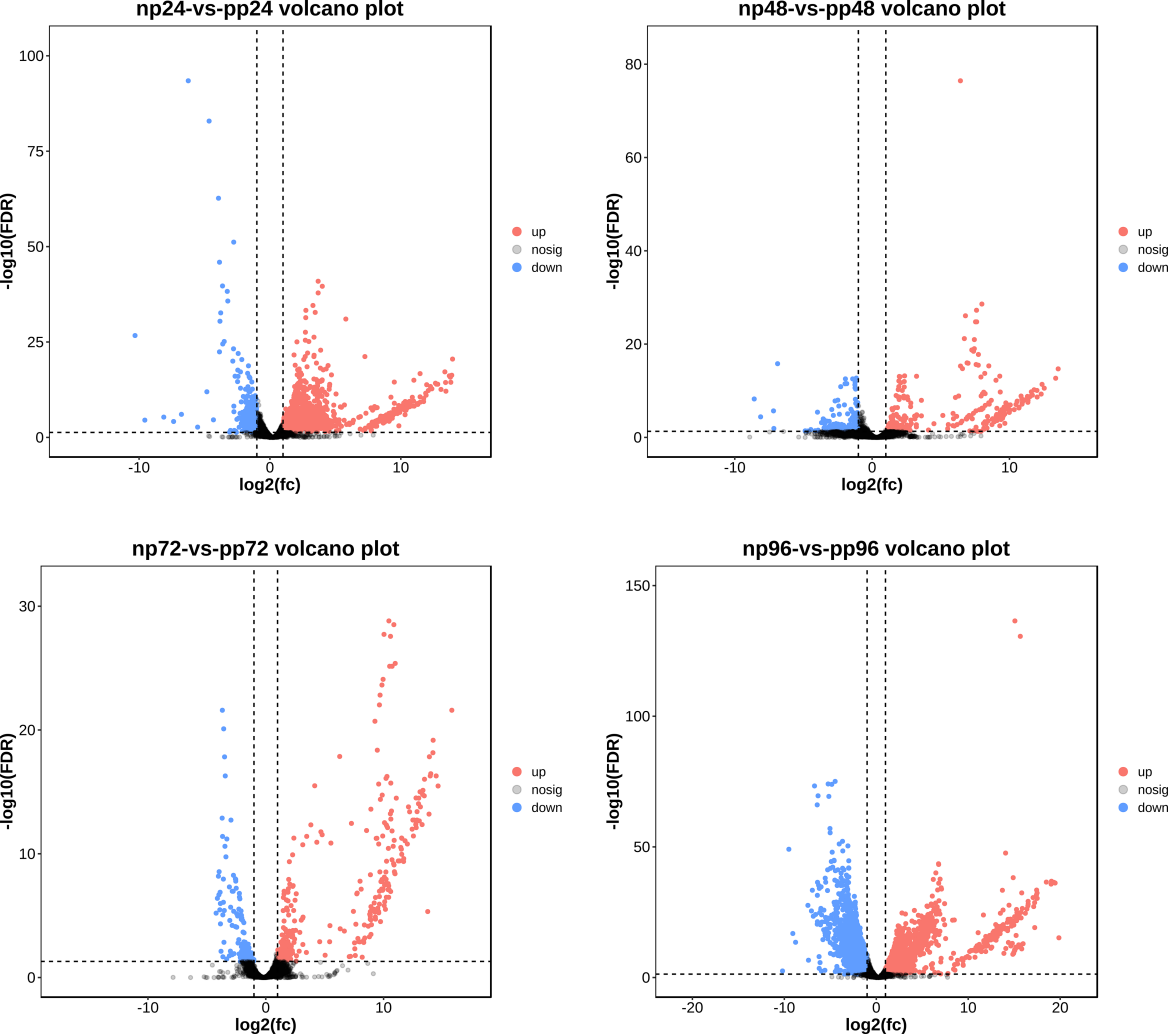
Volcano plots of DEGs during (B) longissima larval parasitization by (A) hispinarum*at* different time points. **(A)** Volcano plot of DEGs at 24 h post-parasitization (np24-vs-pp24). **(B)** Volcano plot of DEGs at 48 h post-parasitization (np48-vs-pp48). **(C)** Volcano plot of DEGs at 72 h post-parasitization (np72-vs-pp72). **(D)** Volcano plot of DEGs at 96 h post-parasitization (np96-vs-pp96). In each volcano plot, the x-axis represents the log_2_(fold change) (log_2_FC) of gene expression, and the y-axis represents the -log_10_(false discovery rate) (-log_10_FDR). Red dots indicate significantly upregulated DEGs, blue dots indicate significantly downregulated DEGs, and gray dots indicate non-significant DEGs (nosig). The vertical dashed lines denote the threshold for |log_2_FC|, and the horizontal dashed line denotes the threshold for -log_10_(FDR) (typically corresponding to an FDR < 0.05).

Volcano plots were used to visualize the overall distribution of DEGs in each comparison group. In these plots, significantly upregulated genes are shown as red dots, significantly downregulated genes as blue dots, and genes without significant expression changes as grey dots. The volcano plots for all comparisons are presented in **Figure 7**. Specifically, the np24 vs. pp24 comparison revealed 2,120 DEGs, including 1,624 upregulated and 496 downregulated genes. In the np48 vs. pp48 comparison, 467 DEGs were detected, with 286 genes upregulated and 181 downregulated. The np72 vs. pp72 comparison identified 500 DEGs, comprising 341 upregulated and 159 downregulated genes. The largest number of DEGs was observed in the np96 vs. pp96 comparison, with 5,749 DEGs, of which 3,332 were upregulated and 2,417 were downregulated. Overall, across all four time points, the majority of differentially expressed genes showed an upregulation trend following parasitism (**Figure 7**).

#### 3.2.2 Functional annotation and enrichment analysis of differentially expressed genes

To further clarify the biological functions of the differentially expressed genes (DEGs), Gene Ontology (GO) enrichment analysis was conducted for each comparison between parasitized and non-parasitized larvae at different time points. The top 20 GO terms enriched by DEGs, particularly those associated with immune responses to parasitism, are presented in the corresponding figures.

**Figure 8:**
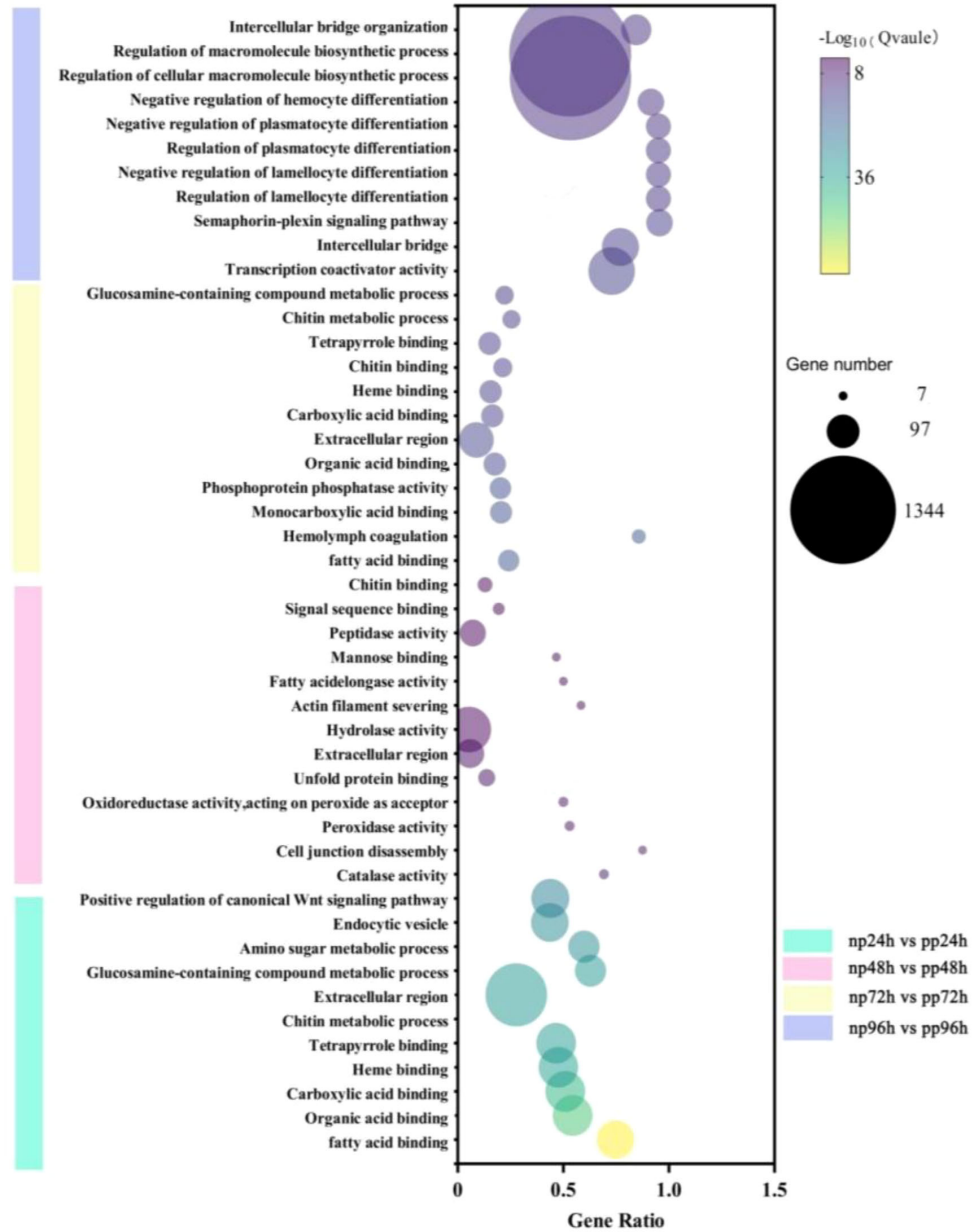
Top 20 GO pathway enrichments of DEGs at different parasitism time points in (B) longissima larvae parasitized by (A) hispinarum(related to parasite immunity) The bubble plot illustrates the enrichment of differentially expressed genes (DEGs) in the top 20 Gene Ontology (GO) pathways associated with parasite immunity. The x-axis represents the gene ratio (proportion of DEGs in a pathway), the y-axis lists the GO terms (sorted by significance), and the size of each bubble corresponds to the number of DEGs in the pathway (key: 7, 97, 1344 genes). The color gradient (from purple to yellow) indicates the -log₁₀(Q-value) (significance level, with higher values indicating stronger enrichment). Different colors represent different time points: np24-vs-pp24 (cyan), np48-vs-pp48 (pink), np72-vs-pp72 (yellow), and np96-vs-pp96 (purple).

GO enrichment analysis revealed dynamic and stage-specific functional responses following parasitism. At 24 h (np24 vs. pp24), DEGs were predominantly enriched in binding-related functions, including fatty acid binding, organic acid binding, and heme binding, along with cellular component terms such as extracellular region and endocytic vesicle. At 48 h (np48 vs. pp48), enrichment shifted toward hydrolase-related activities, including catalase, peroxidase, and peptidase activities, as well as processes associated with cell adhesion and structural remodeling, such as cell junction disassembly and actin filament severing. By 72 h (np72 vs. pp72), DEGs were mainly involved in immune and metabolic processes, including hemolymph coagulation, peptidase activity, glucosamine-containing compound metabolism, and continued enrichment in binding functions and extracellular components. At 96 h (np96 vs. pp96), enrichment was strongly associated with immune regulation and hemocyte differentiation, including pathways related to the regulation of lamellocyte and plasmatocyte differentiation, semaphorin–plexin signaling, intercellular bridge organization, and transcription coactivator activity, indicating a pronounced late-stage immune response (**Figure 8**).

Overall, these results indicate that parasitism by *A. hispinarum* induces dynamic and time-dependent changes in gene expression in *B. longissima*, with early responses dominated by binding and metabolic functions and later responses strongly associated with immune cell differentiation and regulation.

Through KEGG pathway enrichment analysis, we investigated the functional significance of differentially expressed genes (DEGs) at various time points following parasitism. At 24 hours post-parasitism, DEGs were mapped to 315 KEGG pathways, of which 25 were significantly enriched (Q value < 0.05). Key enriched pathways included protein digestion and absorption, the PI3K-Akt signaling pathway, and ECM–receptor interaction, among others (**Table 2**).

**Table 2:** The KEGG significant enrichment pathway of 24 h in *B. longissima* larva following parasitization by *A. hispinarum*.

Pathway	np24-vs-pp24 (799)	Pvalue	Pathway ID
ECM-receptor interaction	71	2.48E-22	ko04512
Amoebiasis	70	1.05E-19	ko05146
AGE-RAGE signaling pathway in diabetic complications	47	9.74E-19	ko04933
Protein digestion and absorption	46	9.45E-17	ko04974
Amino sugar and nucleotide sugar metabolism	47	2.71E-16	ko00520
Relaxin signaling pathway	46	1.50E-13	ko04926
Small cell lung cancer	50	4.44E-13	ko05222
PI3K-Akt signaling pathway	83	7.54E-11	ko04151
Human papillomavirus infection	102	5.29E-08	ko05165
Inflammatory bowel disease	6	1.22E-05	ko05321
Focal adhesion	77	1.76E-05	ko04510
Pathways in cancer	84	1.91E-05	ko05200
Antigen processing and presentation	29	2.81E-05	ko04612
IL-17 signaling pathway	17	2.97E-05	ko04657
Cytosolic DNA-sensing pathway	8	2.99E-05	ko04623
Complement and coagulation cascades	10	8.61E-05	ko04610
Protein export	11	0.00013317	ko03060
DNA replication	10	0.000271738	ko03030
Lipid and atherosclerosis	37	0.000483485	ko05417
Prostate cancer	23	0.000506222	ko05215
Cell cycle	26	0.001280773	ko04110
Arrhythmogenic right ventricular cardiomyopathy	20	0.002468712	ko05412
Acute myeloid leukemia	13	0.002855724	ko05221
B cell receptor signaling pathway	11	0.003213767	ko04662
Glycosaminoglycan biosynthesis - heparan sulfate/heparin	7	0.003867367	ko00534

At 48 hours post-parasitism, DEGs were associated with 252 KEGG pathways, with 22 pathways showing significant enrichment. These included apoptosis, protein processing in the endoplasmic reticulum, and lysosome-related pathways (**Table 3**).

**Table 3:** The KEGG significant enrichment pathway of 48 h in *B. longissima* larva following parasitization by *A. hispinarum*.

Pathway	np48-vs-pp48 (217)	Pvalue	Pathway ID
Antigen processing and presentation	23	4.992722e-13	ko04612
Fatty acid elongation	10	8.461927e-09	ko00062
Protein processing in endoplasmic reticulum	28	4.216645e-07	ko04141
Lysosome	29	1.232821e-05	ko04142
Apoptosis	19	2.678867e-05	ko04210
Spliceosome	16	0.000144102	ko03040
Estrogen signaling pathway	13	0.000209839	ko04915
Rheumatoid arthritis	11	0.000221608	ko05323
Biosynthesis of unsaturated fatty acids	7	0.000260149	ko01040
Longevity regulating pathway - worm	11	0.000441022	ko04212
Legionellosis	10	0.000531867	ko05134
Lipid and atherosclerosis	15	0.000694034	ko05417
Fatty acid metabolism	18	0.000726952	ko01212
Necroptosis	10	0.000833622	ko04217
IL-17 signaling pathway	7	0.001087392	ko04657
NOD-like receptor signaling pathway	10	0.001142346	ko04621
Autophagy - animal	16	0.001833368	ko04140
Fluid shear stress and atherosclerosis	12	0.002094725	ko05418
Nucleocytoplasmic transport	14	0.003001381	ko03013
Prostate cancer	9	0.003483324	ko05215
Progesterone-mediated oocyte maturation	8	0.004077558	ko04914
Th17 cell differentiation	6	0.004235618	ko04659

By 72 hours post-parasitism, 199 KEGG pathways were enriched, 23 of which were significant. The primary pathways involved the PI3K-Akt signaling pathway, ECM–receptor interaction, and DNA replication (**Table 4**).

**Table 4:** The KEGG significant enrichment pathway of 72 h in *B. longissima* larva following parasitization by *A. hispinarum*.

Pathway	np72-vs-pp72 (172)	Pvalue	Pathway ID
Complement and coagulation cascades	18	7.747894e-28	ko04610
Coronavirus disease - COVID-19	20	5.617284e-13	ko05171
Neutrophil extracellular trap formation	18	8.956681e-12	ko04613
ECM-receptor interaction	23	1.679352e-10	ko04512
Antigen processing and presentation	18	3.132207e-10	ko04612
PI3K-Akt signaling pathway	30	3.020534e-09	ko04151
Platelet activation	20	3.037193e-08	ko04611
DNA replication	8	1.01616e-07	ko03030
Cell cycle	14	1.71371e-06	ko04110
Progesterone-mediated oocyte maturation	9	0.000181895	ko04914
Protein processing in endoplasmic reticulum	19	0.000279846	ko04141
Human papillomavirus infection	27	0.000292203	ko05165
Focal adhesion	23	0.000303153	ko04510
Estrogen signaling pathway	11	0.000381329	ko04915
Legionellosis	9	0.000410498	ko05134
Spliceosome	13	0.000492249	ko03040
Lipid and atherosclerosis	13	0.000694815	ko05417
Nucleocytoplasmic transport	13	0.001026759	ko03013
Rheumatoid arthritis	8	0.002926639	ko05323
Fluid shear stress and atherosclerosis	10	0.003457077	ko05418
Riboflavin metabolism	3	0.003498871	ko00740
Amyotrophic lateral sclerosis	18	0.004531599	ko05014
Longevity regulating pathway - worm	8	0.004725637	ko04212

**Figure 9:**
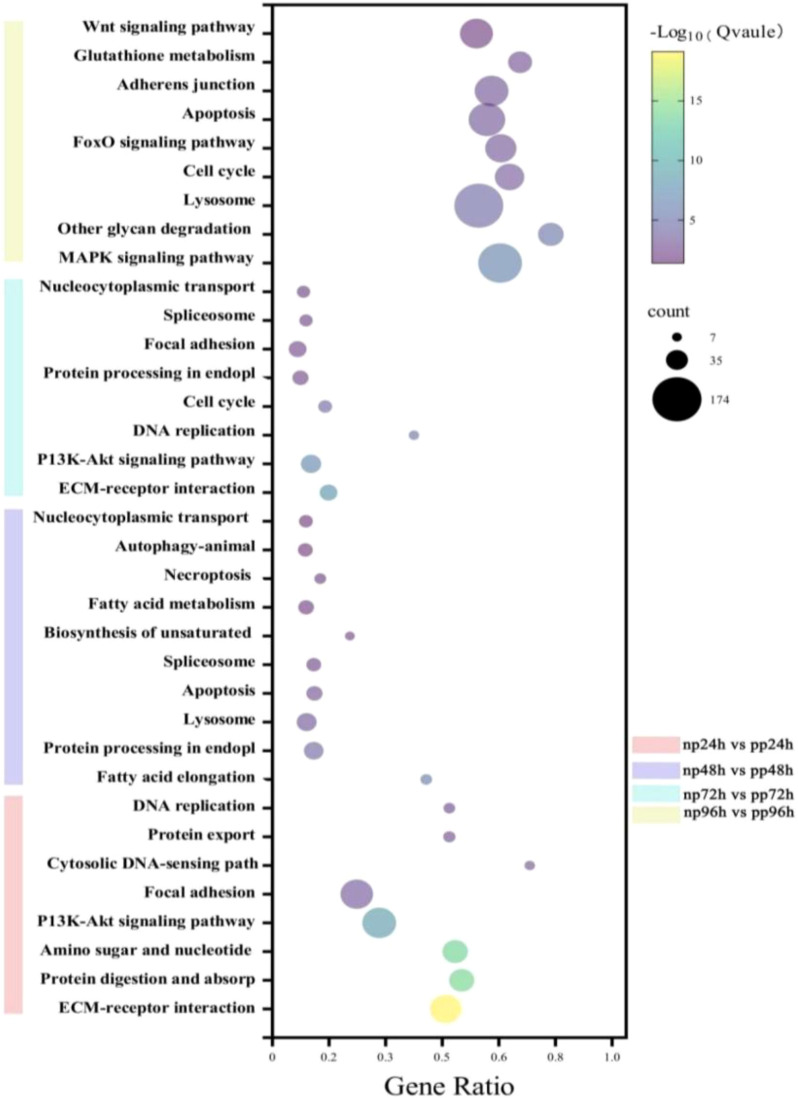
Top 20 KEGG pathway enrichments of DEGs at different parasitism time points in (B) longissima larvae parasitized by (A) hispinarum(related to parasite immunity) The bubble plot illustrates the enrichment of differentially expressed genes (DEGs) in the top 20 Kyoto Encyclopedia of Genes and Genomes (KEGG) pathways associated with parasite immunity. The x-axis represents the gene ratio (proportion of DEGs in a pathway), the y-axis lists the KEGG terms (sorted by significance), and the size of each bubble corresponds to the number of DEGs in the pathway (key: 7, 35, 174 genes). The color gradient (from purple to yellow) indicates the -log₁₀(Q-value) (significance level, with higher values indicating stronger enrichment). Different colors represent different time points: np24-vs-pp24 (pink), np48-vs-pp48 (purple), np72-vs-pp72 (cyan), and np96-vs-pp96 (yellow).

At 96 hours post-parasitism, DEGs were enriched in 338 KEGG pathways, with 20 significantly enriched. Notable pathways included the MAPK signaling pathway, Wnt signaling pathway, cell cycle regulation, and lysosome-related processes (**Table 5**; **Figure 9**).

**Table 5:** The KEGG significant enrichment pathway of 96 h in *B. longissima* larva following parasitization by *A. hispinarum*.

Pathway	np96-vs-pp96 (2326)	Pvalue	Pathway ID
MAPK signaling pathway	139	1.138187e-09	ko04010
Proteoglycans in cancer	165	2.832244e-09	ko05205
Salmonella infection	156	4.40654e-09	ko05132
Other glycan degradation	48	3.708782e-08	ko00511
Lysosome	175	3.538883e-07	ko04142
Prostate cancer	54	9.396751e-07	ko05215
Antigen processing and presentation	61	2.833277e-06	ko04612
Cell cycle	63	6.26788e-06	ko04110
Complement and coagulation cascades	17	9.922283e-06	ko04610
FoxO signaling pathway	71	1.251356e-05	ko04068
Apoptosis	97	1.844616e-05	ko04210
Adherens junction	83	2.419683e-05	ko04520
Parathyroid hormone synthesis, secretion and action	52	2.93376e-05	ko04928
Glutathione metabolism	42	4.68079e-05	ko00480
Renin-angiotensin system	29	0.000129787	ko04614
Breast cancer	50	0.000481758	ko05224
Wnt signaling pathway	79	0.000997078	ko04310
Morphine addiction	26	0.001225022	ko05032
Notch signaling pathway	31	0.001281882	ko04330
Longevity regulating pathway - multiple species	42	0.001767213	ko04213
Bladder cancer	24	0.003172305	ko05219
Cellular senescence	54	0.003593351	ko04218
Longevity regulating pathway	46	0.004363872	ko04211
MicroRNAs in cancer	113	0.004491494	ko05206

These findings highlight key signaling and metabolic pathways that are dynamically regulated in *B. longissima* following A. hispinarum parasitism. Understanding these enriched pathways provides valuable insights into the molecular mechanisms by which parasitism suppresses the immune response of B. *longissima* larvae.

#### 3.2.3 Impact of parasitism on host immune-related gene expression

Sequencing analysis suggested that parasitism by *Asecodes hispinarum* affects the immune system of *Brontispa longissima*, prompting a detailed examination of immune-related genes (**Supplementary Table 2**). In insects, the first step of the immune response is recognition, mediated by pattern recognition receptors (PRRs). In this study, PRRs such as beta-1,3-glucan recognition proteins (βGBP) and Down syndrome cell adhesion molecules (Dscam) were upregulated at 24 h and 48 h post-parasitism. Additionally, βGBP expression remained elevated at 96 h.

Regulatory genes, which modulate immune responses, also showed dynamic changes. Serine protease persephone and serine protease inhibitor dipetalogastin were downregulated following parasitism, while serine protease inhibitor 28Dc-like was upregulated during the early stages of parasitism.

Key components of the melanization pathway were differentially expressed: prophenoloxidase activating factor (PPAF) was upregulated at 24 h, phenoloxidase expression was downregulated at 72 h and 96 h, and PPAF was again upregulated at 96 h. These results suggest that *A. hispinarum* suppresses the immune response of *B. longissima* larvae by inhibiting the conversion of prophenoloxidase into active phenoloxidase.

Furthermore, major insect immune signaling pathways, including Toll, IMD (immune deficiency), JAK/STAT (Janus kinase/signal transducer and activator of transcription), and MAPK (mitogen-activated protein kinase), were affected by parasitism. The expression of genes associated with these pathways was modulated over the course of parasitism, indicating that *A. hispinarum* manipulates multiple immune signaling mechanisms to suppress host defense responses.

These findings highlight the complex and time-dependent modulation of the host immune system by parasitic wasps, providing valuable insights into host–parasite interactions at the molecular level.

#### 3.2.4 qRT-PCR validation of transcriptomic data

**Figure 10:**
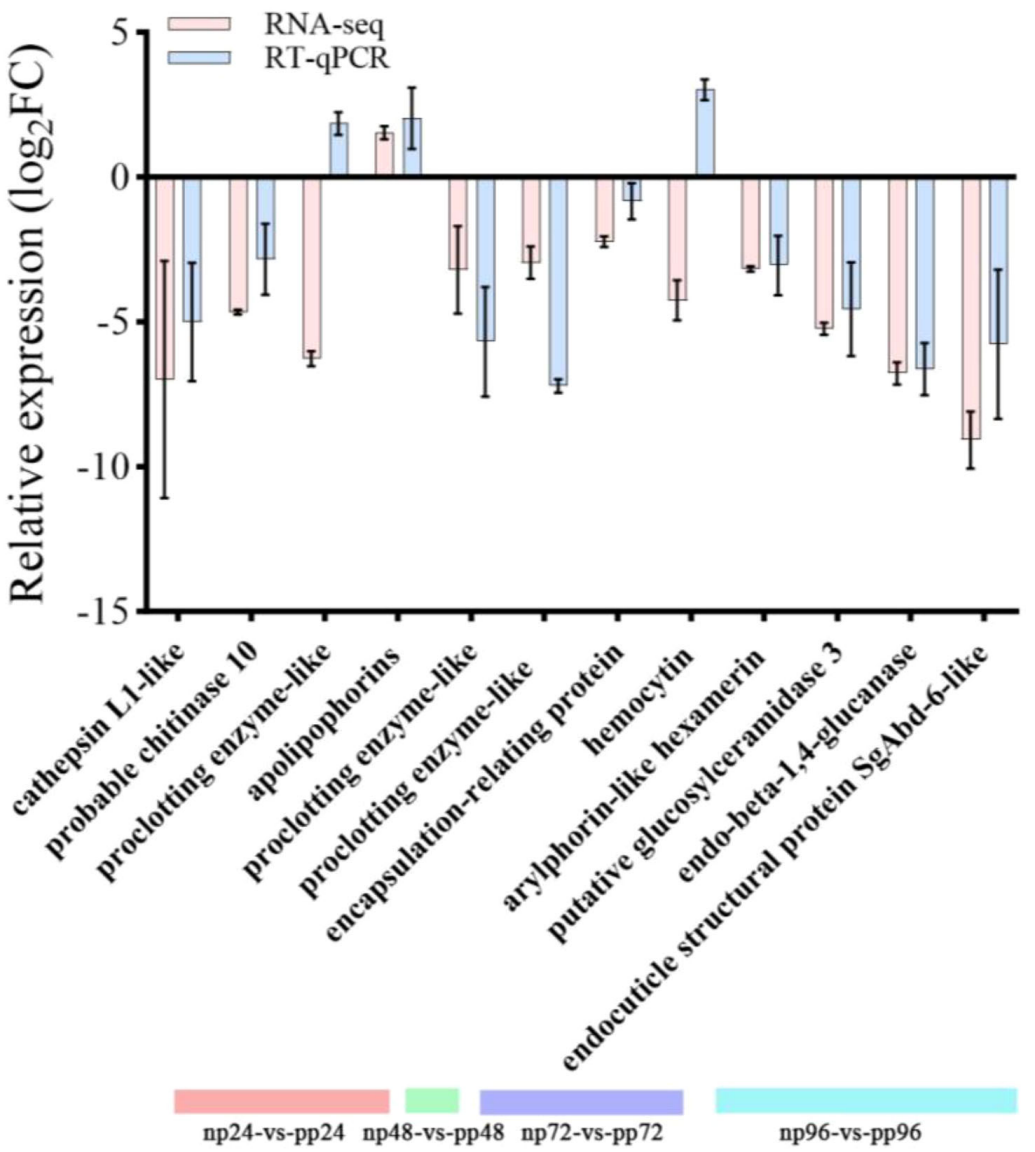
qRT - PCR validation of 12 selected genes in (B) longissima larvae with differential expression after parasitization by (A) hispinarum (based on Illumina sequencing analysis). The relative expression levels of these unigenes were transformed into the log_2_(ratio) of parasitized (P) to non - parasitized (NP). In the bar graph, the red bars represent the RNA - seq data, and the blue bars represent the RT - qPCR data. Different colors at the bottom (red, green, purple, cyan) correspond to different time - point comparisons: np24 - vs - pp24, np48 - vs - pp48, np72 - vs - pp72, and np96 - vs - pp96, respectively. The error bars indicate the standard deviation (or standard error) of the mean values from independent biological replicates.

To confirm the reliability of the Illumina-based transcriptomic analysis, twelve genes were randomly selected for validation using quantitative real-time PCR (qRT-PCR). The results showed that ten of the twelve genes exhibited expression patterns consistent with the RNA-seq data, reflecting similar trends of upregulation or downregulation in response to parasitism. However, two genes displayed discrepancies between qRT-PCR and Illumina sequencing results (**Figure 10**).

Overall, these findings support the accuracy and reproducibility of the transcriptomic analysis, while also highlighting the importance of experimental validation for individual genes.

## 4 Discussion

Parasitic wasps are a diverse and ecologically significant group within the order Hymenoptera. These wasps are capable of manipulating multiple physiological processes in their host insects, including immunity, metabolism, and development, often leading to the host’s death (22–24). As such, they serve as critical natural biocontrol agents and are widely recognized as effective tools for biological pest management.

Insects, through long evolutionary histories, have developed a robust innate immune system consisting of both humoral and cellular components. Humoral immunity is primarily mediated through three key signaling pathways: Toll, IMD (immune deficiency), and JAK/STAT (Janus kinase/signal transducer and activator of transcription). These pathways regulate the expression of immune-related genes via signal transduction cascades, ultimately inducing the production of antimicrobial peptides and other effector molecules (25, 26). Cellular immunity, mediated by hemocytes, is responsible for pathogen encapsulation, phagocytosis, and aggregation (27, 28).

To comprehensively understand immune modulation by parasitic wasps, it is essential to establish an extensive immune gene database for insects, which would allow for more accurate classification of immune-related genes and proteins. However, current databases such as KEGG, GO, and GOG do not provide a dedicated classification for insect immune genes (29). Research on the immune system of *Brontispa longissima* remains limited. To address this gap, we performed extensive annotation and screening of immune-related genes in *B. longissima* larvae following parasitism by *Asecodes hispinarum*, using homologous sequence alignment and KEGG database information.

Our analysis revealed significant changes in pathogen recognition, signal transduction, regulatory processes, and the expression of effector genes in *B. longissima* larvae after parasitism. These results indicate that *A. hispinarum* parasitism can indeed activate and modulate the immune response of *B. longissima*.

Recognition is the first step in the insect immune response. Pathogen invasion triggers the activation of pattern recognition receptors (PRRs) and downstream signaling pathways such as Toll and IMD (30, 31). PRRs serve multiple roles: they act as pathogen sensors, opsonins that promote phagocytosis, and initiators of immune signaling cascades (32). In our study, parasitism induced transcriptional regulation of PRRs in *B. longissima* larvae. For example, beta-1,3-glucan-binding protein-like (βGBP) was upregulated at 24 and 48 h, while the Down syndrome cell adhesion molecule-like protein Dscam2 was upregulated at 24 and 96 h. Similar observations have been reported in other host–parasitoid systems. For instance, Trichopria drosophilae parasitism alters the expression of PRRs in Drosophila melanogaster, with PGRP-SA upregulated and PGRP-SB2 and CG9673 downregulated (33). In Ostrinia furnacalis larvae parasitized by Macrocentrus cingulum, PRRs such as C-type lectins, βGBP, and integrins were significantly upregulated (34).

GO pathway enrichment analysis indicated that hemocyte differentiation-related pathways were enriched at 96 h post-parasitism, including regulation of lamellocyte and plasmatocyte differentiation and negative regulation of hemocyte differentiation. In insects, hemocyte quantity and function can be affected by foreign invaders. For example, in *Helicoverpa armigera*, total hemocyte counts remain unchanged after *Nomuraea rileyi* infection, yet phagocytosis, nodulation, and encapsulation are impaired (35). A similar phenomenon was observed in *B. longissima*, where the proportion of plasmatocytes remained largely unchanged, and encapsulation responses were absent. Plasmatocytes are the primary hemocyte type responsible for cellular immunity, pathogen engulfment, and encapsulation (36, 37). In *Bombyx mori*, plasmatocytes can differentiate into oenocytoids, which contain prophenoloxidase (PPO) precursors and participate in melanization, humoral immunity, and encapsulation. Lamellocytes, on the other hand, specialize in encapsulating parasitoids and large foreign objects (38).

In our study, the transcription of prophenoloxidase activating factor (PPAF) and phenoloxidase (PO) was upregulated at 24 h post-parasitism, but PO expression was suppressed at 72 and 96 h. This may relate to the negative regulation of plasmatocyte differentiation. Typically, insect immune responses involve melanization alongside encapsulation (39–41). Melanization is a serine protease (SP)-mediated cascade, where inactive PPO is converted to active PO by terminal PPAFs (24). Here, serine protease persephone was downregulated at 24, 48, and 72 h, which aligns with its role in Toll pathway activation in *Drosophila*. Serine protease inhibitor dipetalogastin was downregulated at 24 and 48 h, consistent with its antibacterial and immune-regulatory roles in *Galleria mellonella* (42). Activation of the PO cascade not only drives melanization but also supports coagulation, nodule formation, and encapsulation (43). Parasitic wasps are known to disrupt this cascade: for example, LbSPNy in Leptopilina boulardi venom inhibits PO activation in *D.* melanogaster (44), and Scleroderma guani suppresses melanization in Tenebrio molitor (45). Comparable effects have been reported in Octodonta nipae and *O. nipae* pupae parasitized by Tetrastichus brontispae (40).

Overall, *A. hispinarum* parasitism induces widespread modulation of both humoral and cellular immunity in *B. longissima* larvae. While these findings reveal critical immune responses, the underlying mechanisms remain incompletely understood and warrant further investigation.

## 5 Conclusions

This study presents the first comprehensive transcriptome of *B. longissima* larvae and provides a pioneering analysis of how *A. hispinarum* parasitism regulates immune-related gene expression. Comparative transcriptomic analysis revealed that parasitism modulates genes involved in pathogen recognition, signal transduction, regulatory processes, and melanization, thereby disrupting both cellular and humoral immunity. These immune-related genes provide insight into the molecular mechanisms by which parasitic wasps suppress host defenses. Furthermore, they offer candidate targets for developing pest control strategies based on manipulation of host immunity and contribute to a better understanding of host–parasitoid interactions at the molecular level.

## Data Availability

The raw sequence data reported in this paper have been deposited in the Genome Sequence Archive in National Genomics Data Center, China National Center for Bioinformation / Beijing Institute of Genomics, Chinese Academy of Sciences (GSA: CRA041259, CRA041261) that are publicly accessible at https://ngdc.cncb.ac.cn/gsa/browse/CRA041261, https://ngdc.cncb.ac.cn/gsa/browse/CRA041259.
