# Supplementary material for: Comprehensive transcriptomic analysis reveals immune response modulation in Brontispa longissima Gestro larvae following parasitism by Asecodes hispinarum Bouček
Source: Front Immunol. 2026 May 12;17:1823349. doi: 10.3389/fimmu.2026.1823349 (PMC13201176; doi:10.3389/fimmu.2026.1823349)
Supplement: Supplementary file 1 [file Table1.pdf]

Supplementary Material

1 Supplementary Tables

Table S1. Primers used for qRT-PCR validation of selected differentially expressed genes (DEGs) in *B. longissima* larvae after parasitization by *A. hispinarum*.

Gene Name	Forward Primer(5'-3')	Reverse Primer(5'-3')
hemocytin	GACGATAAGACGTGCGTTAG	CTGTAGTTGTGCAGACAGATT
endocuticle structural protein SgAbd-6-like	CGCTGCTTTCATAGCATTTAC	TTGCACGACAGCTTCTTC
arylphorin-like hexamerin	CCCAGGCTGATCCTAATTTC	GAATGGCATGGTGATATGAAAG
putative glucosylceramidase 3	GGCACGTTTACGGATTCA	CCGTGAGGTTTACATCGTTAG
endo-beta-1,4-glucanase	ATTTACAGGTGGCGAAGATAC	CCCGGAAGTGCAATATCAA
probable chitinase 10	CCAAGGGTTTCTGCGATTA	TTCCCACTGGTGTTGTTG
proclotting enzyme-like	ACCGCCTCCTTCAAATAATC	GGTAATGGCGGTATGGTATG
apolipophorins	CTAAAGCAAGAGCCCGTAAA	TATTCCCATAGGGCTGGATTA
cathepsin L1-like	CCTTACTGTGCCAGACTTTAT	GGGCACTCAGGGAAATAAG
Gapdh	TGTCGTATCTTCCGACTTTATC	GGAGGCGGCATACTTAATC
RpL5	GCTTTCAAGAAACAGTTCAGTC	GCTTCCTTCTGTTCCATCTC
phenoloxidase-2	GGCACTGGCATTTAGTGTAT	GCTCGACACGTTTCATCTT
phenoloxidase-3	TTGGCGGTAGCGATTTATG	TCTGGAAAGCTGTGAACTATG
