# Supplementary material for: Comprehensive transcriptomic analysis reveals immune response modulation in Brontispa longissima Gestro larvae following parasitism by Asecodes hispinarum Bouček
Source: Front Immunol. 2026 May 12;17:1823349. doi: 10.3389/fimmu.2026.1823349 (PMC13201176; doi:10.3389/fimmu.2026.1823349)
Supplement: Supplementary file 2 [file Table2.pdf]

Supplementary Material

1 Supplementary Tables

Table S2-1. Immune-related genes differentially transcribed in *B. longissima* larva following parasitization by *A. hispinarum after 24h*

Gene function	Gene description	Blast species	log2(FoldChange)	FDR	Regulate
Recognition	beta-1,3-glucan-binding protein-like	*Anoplophora glabripennis*	1.7571	0.000138552525696027	UP
	Down syndrome cell adhesion molecule-like protein Dscam2 isoform X12	*Leptinotarsa decemlineata*	1.4859	0.00107003933127054	UP
Modulator	serine protease persephone	*Anoplophora glabripennis*	-1.0508	0.0254814970959897	DOWN
	serine protease inhibitor dipetalogastin	*Anoplophora glabripennis*	-1.8667	2.36296652339809E-13	DOWN
	modular serine protease-like	*Leptinotarsa decemlineata*	1.1349	0.00802972768705216	UP
	serine protease inhibitor 28Dc-like	*Leptinotarsa decemlineata*	1.6124	8.92368284132108E-06	UP
	serine protease 44-like	*Leptinotarsa decemlineata*	1.7565	0.0000162387175066543	UP
melanization	prophenoloxidase activating factor 3	*Octodonta nipae*	1.7480	0.0000159365712404408	UP
	phenoloxidase-activating factor 2-like	*Diabrotica virgifera virgifera*	1.0708	0.00191947921384094	UP
Toll and Imd signaling pathway	protein toll	*Anoplophora glabripennis*	-1.1915	5.81723569558341E-06	DOWN
JAK-STAT signaling pathway	G1/S-specific cyclin-D3-like	*Anoplophora glabripennis*	-1.6835	0.0000000005244260372	DOWN
MAPK signaling pathway	bromodomain protein	*Gregarina niphandrodes*	2.5984	0.000879182361239561	UP
	CREB-binding protein	*Anoplophora glabripennis*	1.0790	0.0378410808	UP

isoform X1			861211	
histone acetyltransferase p300 isoform X5	*Nasonia vitripennis*	9.3794	1.9739184099 4576E-06	UP
phosphatidylinositol 3- and 4-kinase	*Gregarina niphandrodes*	4.8795	0.0010461847 7677021	UP
REDICTED: serine/threonine-protein kinase NLK isoform X1	*Tribolium castaneum*	-1.1908	0.0000638029 294641335	DOWN
PREDICTED: serine/threonine-protein kinase NLK isoform X2	*Tribolium castaneum*	-1.3381	7.1415338838 4793E-06	DOWN
GRB2-associated-binding protein 1-like	*Diabrotica virgifera virgifera*	1.2471	0.0018400617 7656749	UP
S phase cyclin A-associated protein in the endoplasmic reticulum isoform X2	*Anoplophora glabripennis*	-2.4734	2.4118444391 3078E-15	DOWN
protein kinase domain protein, partial	*Gregarina niphandrodes*	4.2327	0.0006119571 55952549	UP
protein kinase	*Gregarina niphandrodes*	2.8499	0.0367150631 284699	UP
M-phase inducer phosphatase isoform X3	*Nasonia vitripennis*	10.4710	1.3669424428 3472E-09	UP
heat shock protein	*Phenacoccus solenopsis*	12.8218	1.1613007750 0786E-14	UP
heat shock protein 70	*Gregarina niphandrodes*	3.2096	4.7511932661 3858E-06	UP
PREDICTED: profilin	*Ceratosolen solmsi marchali*	10.3238	1.1420233911 9291E-06	UP

Table S2-2. Immune-related genes differentially transcribed in *B. longissima* larva following parasitization by *A. hispinarum after* 48h

Gene function	Gene description	Blast species	log2(FoldChange)	FDR	Regulate
Recognition	beta-1,3-glucan-binding protein-like	*Anoplophora glabripennis*	1.7779	0.000735322266330628	UP
Modulator	serine protease inhibitor dipetalogastin	*Anoplophora glabripenni*	-1.9343	0.0272637075758652	DOWN
	serine protease inhibitor 28Dc-like	*Leptinotarsa decemlineata*	1.4681	0.0172245004594049	UP
	serine protease persephone	*Anoplophora glabripennis*	-1.3127	0.0000134200575653883	DOWN
Toll and Imd signaling pathway	stress-activated protein kinase JNK isoform X3	*Sitophilus oryzae*	1.4911	0.0021162047688563	UP
JAK-STAT signaling pathway	histone acetyltransferase p300 isoform X5	*Nasonia vitripennis*	4.1028	0.0261159303447429	UP
	phosphatidylinositol 3- and 4-kinase	*Gregarina niphandrodes*	-4.4620	0.0206881899851958	DOWN
MAPK signaling pathway	M-phase inducer phosphatase isoform X3	*Nasonia vitripennis*	9.9189	8.49319049251962E-07	UP
	heat shock protein	*Phenacoccus solenopsis*	7.6015	5.52676762814639E-28	UP
	PREDICTED: profilin	*Ceratosolen solmsi marchali*	12.0154	2.21191527541669E-09	UP
	heat shock protein 70	*Anoplophora glabripennis*	-2.6490	0.00760662333676605	DOWN

Table 2-3. Immune-related genes differentially transcribed in *B. longissima* larva following parasitization by *A. hispinarum after* 72h

**Gene function**	**Gene description**	**Blast species**	**log2(FoldChange)**	**FDR**	**Regulate**
Modulator	serine protease persephone	*Anoplophora glabripennis*	-1.1130	0.0294397050334885	DOWN
melanization	phenoloxidase 2-like	*Leptinotarsa decemlineata*	-3.4970	1.48447884341535E-18	DOWN
	phenoloxidase 1-like	*Sitophilus oryzae*	-3.5971	1.11311657558481E-08	DOWN
Toll and Imd signaling pathway	PREDICTED: transcription factor kayak isoform X2	*Ceratosolen solmsi marchali*	9.3023	0.0000129771454733444	UP
JAK-STAT signaling pathway	histone acetyltransferase p300 isoform X5	*Nasonia vitripennis*	8.9715	0.0000541869898977753	UP
MAPK signaling pathway	M-phase inducer phosphatase isoform X3	*Nasonia vitripennis*	9.6463	1.17409939361347E-06	UP
	heat shock protein	*Phenacoccus solenopsis*	10.7232	7.11652524043096E-26	UP
	PREDICTED: transcription factor kayak isoform X2	*Ceratosolen solmsi marchali*	9.3023	0.0000129771454733444	UP
	vascular endothelial growth factor receptor 1 isoform X4	*Anoplophora glabripennis*	-1.2341	0.0460191541147128	DOWN
	PREDICTED: profilin	*Ceratosolen solmsi marchali*	12.7700	7.76706655263763E-13	UP

Table S2-4. Immune-related genes differentially transcribed in *B. longissima* larva following parasitization by *A. hispinarum after* 96h

**Gene function**	**Gene description**	**Blast species**	**log2(FoldChange)**	**FDR**	**Regulate**
Recognition	Down syndrome cell adhesion molecule-like protein Dscam2 isoform X12	*Leptinotarsa decemlineata*	2.6355	1.01272152529462E-07	UP
	Down syndrome cell adhesion molecule-like protein Dscam2 isoform X41	*Leptinotarsa decemlineata*	2.5673	1.64865265198788E-07	UP
Modulator	serine protease 44-like	*Leptinotarsa decemlineata*	2.2446	2.49255324832271E-14	UP
melanization	phenoloxidase 1-like	*Sitophilus oryzae*	-3.4286	1.07778742610262E-22	DOWN
	prophenoloxidase activating factor 3	*Octodonta nipae*	2.4325	7.77766753474153E-17	UP
	phenoloxidase 2-like	*Leptinotarsa decemlineata*	-2.1916	4.89369420876843E-21	DOWN
Toll and Imd signaling pathway	ubiquitin-conjugating enzyme E2-17 kDa isoform X1	*Drosophila ananassae*	1.0417	0.0206811030447195	UP
	PREDICTED: transcription factor kayak isoform X2	*Ceratosolen solmsi marchali*	12.4880	2.02195663510035E-15	UP
	protein toll	*Anoplophora glabripennis*	-1.9380	2.28626649256234E-06	DOWN
	probable LRR receptor-like serine/threonine-protein kinase At2g23950	*Anoplophora glabripennis*	-2.1935	3.52297555661305E-08	DOWN
	embryonic polarity protein dorsal-like isoform X1	*Anoplophora glabripennis*	1.1718	0.036620911854043	UP
	serine/threonine-protein kinase pelle-like	*Anoplophora glabripennis*	1.2434	0.00361287418392926	UP
	beta-TrCP isoform X1	*Anoplophora glabripennis*	1.5357	3.7833791900807E-08	UP
	protein toll	*Leptinotarsa decemlineata*	-2.1261	4.59442084424286E-06	DOWN
	ubiquitin-conjugating	*Thrips palmi*	1.0362	0.025736775918	UP

	enzyme E2-17 kDa			9735	
	embryonic polarity protein dorsal-like isoform X1	*Colletes gigas*	10.1557	0.000000000107 2222806	UP
JAK-STAT signaling pathway	phosphatidylinositol 4,5-bisphosphate 3-kinase catalytic subunit delta isoform	*Leptinotarsa decemlineata*	1.6733	0.000037299516 3989845	
	epidermal growth factor receptor	*Leptinotarsa decemlineata*	1.6334	0.000644011812 623579	UP
	bromodomain protein	*Gregarina niphandrodes*	3.8444	5.249939476486 44E-13	UP
	CREB-binding protein isoform X1	*Anoplophora glabripennis*	2.6818	0.000017237434 3738217	UP
	CREB-binding protein isoform X2	*Anoplophora glabripennis*	2.7152	0.000016011858 5704613	UP
	CREB-binding protein isoform X4	*Anoplophora glabripennis*	2.8613	2.009303319148 33E-07	UP
	CREB-binding protein isoform X5	*Anoplophora glabripennis*	2.7374	0.000012294292 6248441	UP
	histone acetyltransferase p300 isoform X5	*Nasonia vitripennis*	12.1108	4.140446383314 92E-13	UP
	phosphatidylinositol 3- and 4-kinase [Gregarina niphandrodes]	*Gregarina niphandrodes*	5.0229	1.130200047100 84E-18	UP
MAPK signaling pathway	heat shock protein	*Phenacoccus solenopsis*	19.0539	1.421634451870 95E-37	UP
	PREDICTED: serine/threonine-protein kinase NLK isoform X1	*Tribolium castaneum*	2.0274	2.423727860046 92E-08	UP
	M-phase inducer phosphatase isoform X3	*Nasonia vitripennis*	10.8434	7.337331615342 1E-11	UP
	protein kinase	*Gregarina niphandrodes*	2.6271	0.003597342890 03811	UP

heat shock protein 70	*Gregarina niphandrodes*	6.0744	1.084815656501 66E-23	UP
protein kinase domain protein, partial	*Gregarina niphandrodes*	4.1657	4.338949961695 22E-12	UP
PREDICTED: profilin	*Ceratosolen solmsi marchali*	15.7515	1.520963240776 5E-23	UP
PREDICTED: transcription factor kayak isoform X2	*Ceratosolen solmsi marchali*	12.4880	2.021956635100 35E-15	UP
PREDICTED: protein groucho-like isoform X2	*Nicrophorus vespilloides*	1.1415	0.004832132246 18373	UP
serine/threonine-protein kinase mig-15 isoform X6	*Anoplophora glabripennis*	2.2180	7.527894879729 85E-15	UP
probable LRR receptor-like serine/threonine-protein kinase At2g23950	*Anoplophora glabripennis*	-2.1935	3.522975556613 05E-08	DOWN

FDR: False discovery rate

Differentially expressed genes were identified on the basis of FDR≤0.05 and the absolute value of log2FC≥1
